# Supplementary material for: Combined Immune Checkpoint Blockade and Helixor® Therapy in Oncology: Real-World Tolerability and Subgroup Survival (ESMO GROW)
Source: Int J Mol Sci. 2025 Apr 12;26(8):3669. doi: 10.3390/ijms26083669 (PMC12027740; doi:10.3390/ijms26083669)
Supplement: Supplementary file 1 [file ijms-26-03669-s001.zip › ijms-3491663-supplementary.pdf]

Supplementary tables and figures

Supplementary Table S1. Characterization of immune checkpoint blockade, total cohort

	CTRL group,n=344	COMB group n=61
Avelumab, n (%)	1 (0.3)	0 (0)
Atezolizumab, n (%)	64 (18.6)	14 (23.0)
Durvalumab, n (%)	19 (5.5)	5 (8.2)
Ipilimumab, n (%)	7 (2.0)	3 (4.9)
Iplimumab/Nivolumab, n (%)	1 (0.3)	0 (0)
Nivolumab, n (%)	46 (13.4)	10 (16.4)
Pembrolizumab, n (%)	205 (59.6)	29 (47.5)
Spartalizumab, n (%)	1 (0.3)	0

Proportion of patients receiving ICB according to groups in the total cohort (n=405); ICB, immune checkpoint blockade

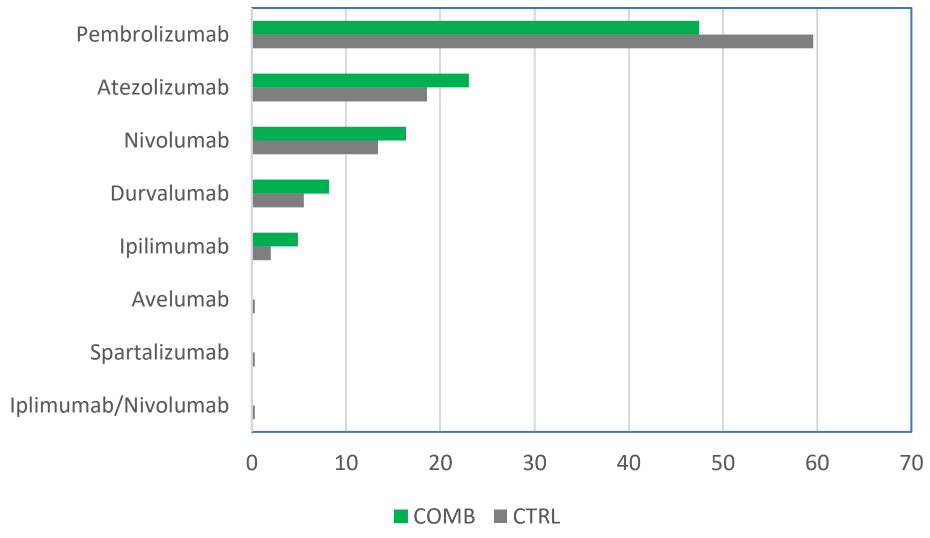

Supplementary Figure S1. Characterization of immune checkpoint blockade, total cohort, n=405

Supplementary Table S2. Factors associated with hazard of death

	aHR	(95% CI)	p-Value
**Combinational ICB+HVA therapy**	**0.088**	**0.009- 0.783**	**0.029***
Chemotherapy	0.548	0.043-6.916	0.642
Radiation	2.800	0.848-9.246	0.091
Surgery	0.533	0.117-2.423	0.416

Cox proportional hazard analysis. Adjusted for standard oncological therapy, stratified for age, tumor stage and first-line therapy. *, $p<0.05$; aHR, adjusted hazard ratio of death; CI, confidence interval

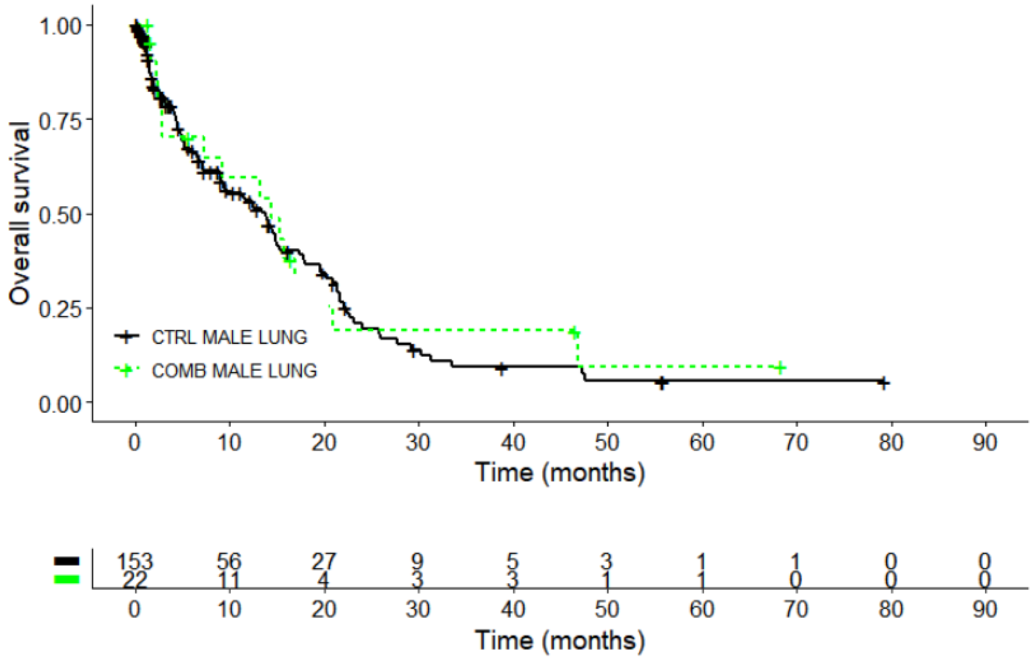

Supplementary Figure S2. Kaplan–Meier survival analysis of overall survival in male NSCLC patients treated with PD-1/PD-L1 inhibitors, with our without Helixor® VA (HVA) therapy (n=179, 4 excluded due to missing data); Log-rank test: $X^2 = 0.1$, p=0.8; CTRL, PD-1/PD-L1 inhibitors COMB, PD-1/PD-L1 inhibitors with HVA therapy; NSCLC, non-small cell lung cancer.

Supplementary Table S3. Median overall survival in male lung cancer patients, n=179.

	N	*Events*	*Median [months]*	*95% CI [months]*
NSCLC male, CTRL	*153*	*94*	*13.8*	*9.33 – 17.4*
NSCLC male, COMB	*22*	*16*	*14.4*	*7.33 - NA*
Log rank test X^2=0.1, p=0.8				

NSCLC, non-small cell lung cancer; X^2, chi-square; p, p-value; CI, confidence interval; CTRL, PD-1/PD-L1 inhibitors COMB, PD-1/PD-L1 inhibitors with HVA therapy.
